# Characterization of t-loop formation by TRF2

**DOI:** 10.1080/19491034.2020.1783782

**Published:** 2020-07-14

**Authors:** Leonid A. Timashev, Titia De Lange

**Affiliations:** Laboratory for Cell Biology and Genetics, Rockefeller University, New York, USA

**Keywords:** Telomere, ATM signaling, TRF2, shelterin, t-loop, Rad51, non-Homologous End Joining, topology

## Abstract

T-loops are thought to hide telomeres from DNA damage signaling and DSB repair pathways. T-loop formation requires the shelterin component TRF2, which represses ATM signaling and NHEJ. Here we establish that TRF2 alone, in the absence of other shelterin proteins can form t-loops. Mouse and human cells contain two isoforms of TRF2, one of which is uncharacterized. We show that both isoforms protect telomeres and form t-loops. The isoforms are not cell cycle regulated and t-loops are present in G1, S, and G2.  Using the DNA wrapping deficient TRF2 Topless mutant, we confirm its inability to form t-loops and repress ATM. However, since the mutant is also defective in repression of NHEJ and telomeric localization, the role of topological changes in telomere protection remains unclear.  Finally, we show that Rad51 does not affect t-loop frequencies or telomere protection. Therefore, alternative models for how TRF2 forms t-loops should be explored.

## Introduction

Telomeres prevent the inappropriate engagement of DNA damage response pathways at the natural ends of linear chromosomes. The telomeric DNA in mammalian cells is associated with a telomere-specific six-subunit protein complex, shelterin, that represses at least six distinct DNA damage response pathways: ATM signaling, ATR signaling, PARP1 activation, Homology Directed Repair (HDR), NHEJ, and alternative Non-Homologous End Joining (alt-NHEJ) [1]. Shelterin is highly compartmentalized with different components of the complex functioning to repress distinct pathways.

TRF2 is one of two double-stranded telomeric DNA binding factors in shelterin [2,3]. TRF2 forms a homodimer allowing two C-terminal Myb/SANT domains to engage the duplex TTAGGG repeat array. TRF2 has the same domain structure as TRF1 but its function is dedicated to the repression of ATM kinase signaling and NHEJ, whereas TRF1 promotes replication of the telomeric DNA [4–9]. TRF2 binds to Rap1 and both TRF1 and TRF2 interact with TIN2 [10–13]. The binding to TIN2 stabilizes TRF1 and TRF2 on telomeres and in addition functions to recruit TPP1 to telomeres [13–15]. TPP1 in turn recruits the sixth subunit of shelterin, POT1, which binds to ss telomeric DNA and represses ATR signaling [16,17]. Upon deletion of TRF2, the residual level of POT1, bound to TRF1-TIN2-TPP1, is sufficient to prevent ATR activation [17]. Conversely, deletion of POT1, TRF1, or TPP1 does not curb the ability of TRF2 to repress ATM signaling and NHEJ [8,17,18].

TRF2 has been proposed to repress ATM signaling and NHEJ by remodeling telomeres into the t-loop structure wherein the ss 3ʹ telomeric end is embedded in the duplex part of the telomere [19,20]. This sequestered telomere terminus is thought to be impervious to the MRN (Mre11/Rad50/Nbs1) DSB sensor in the ATM kinase pathway and prevent the loading of the Ku70/80 initiator of NHEJ. Super-resolution imaging of t-loop frequencies in mouse embryo fibroblasts (MEFs) with and without TRF2 showed that t-loop formation strictly requires TRF2 function [19]. On the other hand, MEFs lacking the mouse POT1 proteins (POT1a and POT1b), Rap1, or TRF1 contain t-loops at normal frequencies [19]. This correlation between t-loop formation by TRF2 and the ability of TRF2 to repress ATM signaling and NHEJ underlies the model that the t-loop structure is a major impediment to activation of these pathways at telomeres. Nonetheless, TRF2 appears to have additional mechanisms to prevent NHEJ that involve its iDDR domain and its interaction with Rap1 [21–23].

While TRF2 was shown to be required for t-loop formation, it has not been established whether TRF2 is sufficient for the remodeling of telomeres. Conceivably, t-loop formation could involve a collaborative effort of TRF2 together with either TIN2 or TPP1 (which have not been tested) or TRF2 could cooperate with multiple shelterin components in a redundant fashion.

The mechanism by which TRF2 remodels telomeres into t-loops is of obvious interest. One model has been proposed based on the finding that the large dimerization domain of TRF2 referred to as the TRFH domain [3,24], weakly binds DNA and can wrap 90 bp around itself [25]. Topological stress induced by DNA wrapping could lead to DNA unwinding which is expected to promote the strand-invasion of the telomere 3ʹ overhang. The wrapping activity of TRF2 requires the presence of seven lysine and two arginine residues on the surface of the TRFH domain [25]. Mutation of these residues to alanine (resulting in the ‘Topless’ mutant of TRF2) abrogates the DNA wrapping activity. TRF2 Topless was shown to be deficient in t-loop formation when expressed in human cells from which the endogenous TRF2 was depleted with shRNA, and under this condition ATM signaling was observed at a subset of the telomeres [25]. Importantly, NHEJ-mediated telomere fusions were not observed, unless Rap1 was removed [25]. These data nominated Topless TRF2 as a separation-of-function mutant and supported the topological model for t-loop formation and repression of ATM kinase activity. Given that these experiments were performed in cells that likely contained residual endogenous TRF2, we sought to further verify the results in a system that allows deletion of TRF2.

We present evidence that the Topless version of TRF2 is deficient in all aspects of telomere protection, suggesting that this is a loss-of-function mutant and therefore not readily interpretable. We show that telomeres containing TRF2 but not the other components of shelterin are present in the t-loop configuration and that both isoforms of TRF2 are equivalent in telomere protection. We also probe the effect of cell cycle stage on t-loop frequencies and examine t-loop formation in absence of Rad51, which has been implicated in t-loop formation using in vitro experiments designed to mimic this process [26]. The results indicate that neither cell cycle aspects nor Rad51 affect the frequency of t-loops at telomeres.

## Results and discussion

### TRF2 is both necessary and sufficient for t-loop formation

**Figure 1.**
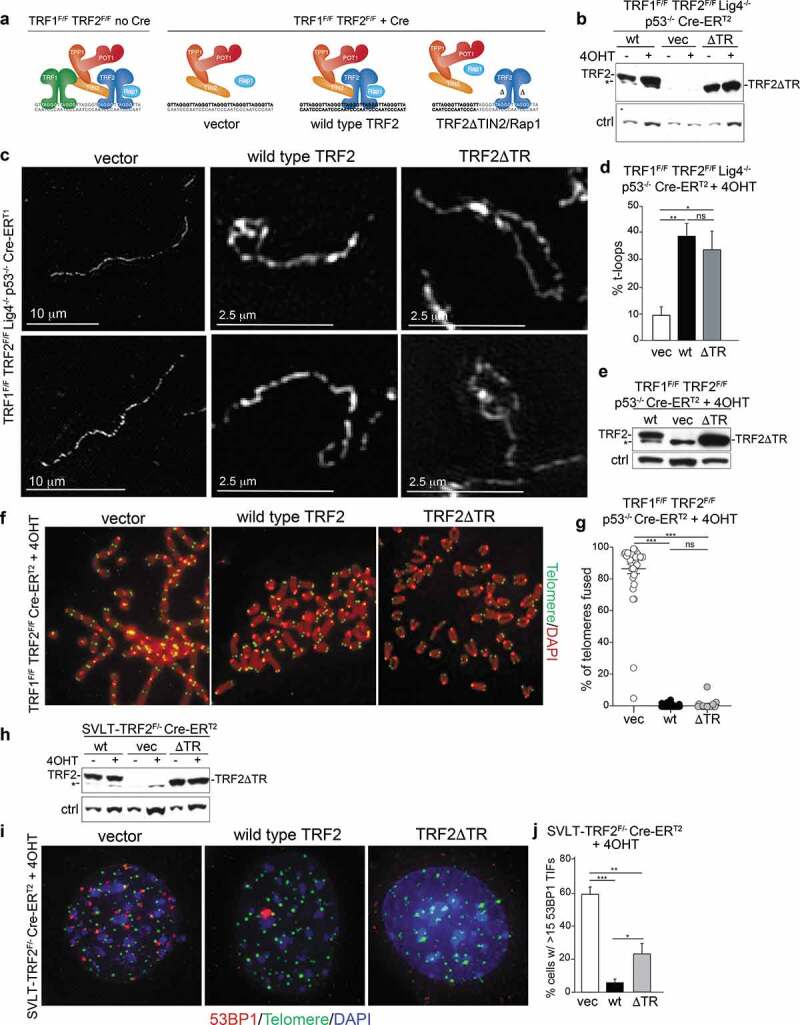
TRF2 is sufficient for the formation of t-loops. (a) Schematic of the experimental approach to create telomeres that contain TRF2 but no other shelterin components. (b) Immunoblot for TRF2 using the indicated MEFs with and without Cre treatment and expressing the indicated versions of TRF2 (wild type, wt; TRF2ΔTR, ΔTR; empty vector, vec). * nonspecific band; ctrl: nonspecific band used as loading control. Note that the endogenous TRF2 is not detected at this exposure. (c) Examples of the structure of telomeric DNA detected in the indicated MEFs treated with Cre (120 h) and infected as in (b). (d) Quantification of t-loop frequencies detected as in (c). Averages and SDs from three independent experiments (>100 molecules scored each). Significance determined using a two-tailed unpaired t-test (n.s. not significant; * p < 0.05; ** p < 0.01). (e) Immunoblots for TRF2 using the indicated MEFs infected with vector, TRF2, and TRF2ΔTR after Cre treatment. * nonspecific band. **f**, Examples of metaphase spreads with telomeres detectedby FISH using the indicated MEFs infected as in (e) and treated with Cre (96 h). (g) Quantification of telomere fusions detected as in (f). Scoring was performed in three independent experiments with 10 metaphase per experiment. Averages, SDs, and significance as in (d). *** p < 0.001. (h) Immunoblot for TRF2 expression in the indicated MEFs infected with vector, TRF2, and TRF2ΔTR before and 96 h after Cre treatment. * nonspecific band. **i**, TIF assay using 53BP1 IF for detection of the telomere damage response on cells as in (h). (j) Quantification of the TIF response as in (i). Averages and SDs from three independent experiments with 50 nuclei each. Significance as above.

In order to test whether TRF2 can form t-loops in the absence of other shelterin components, we made use of TRF1^F/F^ TRF2^F/F^ mouse embryo fibroblasts (MEFs) from which both TRF1 and TRF2 can be deleted by the Cre recombinase [27]. When these cells are provided with exogenous TRF2, the resulting telomeres contain TRF2, Rap1, TIN2, TPP1, and POT1 but no TRF1 [28]. In order to create conditions where the telomeres only contain TRF2, we complemented the cells with an exogenous version of TRF2 that contains deletions of its TIN2 and Rap1 bindings sites (TRF2ΔTIN2ΔRap1 or TRF2ΔTR) [29,30]. Under these conditions, neither TRF1 nor TIN2/TPP1/POT1 or Rap1 are present at the telomeres [27,28] (Figure 1(a)).

In order to determine whether t-loops are formed at telomeres containing only TRF2, we expressed wild type TRF2 and TRF2ΔTR in TRF1^F/F^ TRF2^F/F^ Lig4^-/-^ p53^-/-^ Cre-ER^T2^ MEFs. The use of cells deficient in DNA ligase IV avoids the formation of telomere fusions that confound t-loop analysis [19]. Wild type TRF2 and the TRF2ΔTR were expressed equally before and after Cre treatment and both versions of TRF2 were present in excess over the endogenous TRF2 (Figure 1(b)).

T-loop frequencies were determined using psoralen-UV cross-linked nuclei and the chromatin spreading technique previously established [19] and OMX-imaging of telomeric DNA labeled by FISH [31] (Figure 1(c)). Cells lacking TRF2 (vector control) showed background levels of t-loops, consistent with previous data [19] (Figure 1(c,d)). This t-loop defect was rescued by the expression of wild type TRF2, which is consistent with previous data showing that telomeres lacking TRF1 have normal t-loop levels [19]. The frequency of t-loops achieved by complementation with wild type TRF2 is similar to that of MEFs expressing the endogenous TRF2 (data not shown; see for instance Figure 5). Importantly, there was no difference in t-loop frequency in cells expressing wild type TRF2 and TRF2ΔTR (Figure 1(c,d)). Similarly, TRF2ΔTR was fully proficient in sustaining normal t-loop levels in Cre-treated TRF1^F/F^TRF2^F/F^Ku80^-/-^ MEFs (Supplemental Figure 1(ac)).

Consistent with the ability of TRF2ΔTR to form t-loops, this allele was fully proficient in repressing the formation of telomere fusions when expressed in Lig4-proficient cells (Figure 1(eg)). TRF2ΔTR was also largely proficient in repressing the activation of ATM signaling that occurs in TRF2^F/F^ MEFs treated with Cre (Figure 1(h,i)). The residual DDR response at telomeres containing TRF2ΔTR instead of wild type TRF2 is most likely due to the previously noted minor role of TIN2 in repression of ATM [30].

These data suggest that TRF2 is the only component of shelterin necessary and sufficient for t-loop formation, although it does not exclude the involvement of non-shelterin factors. One caveat in our experiments is that the expression level of the exogenous versions of TRF2 is much greater than the endogenous TRF2 (see Supplemental Figure 1(d)), possibly masking a subtle defect in t-loop formation. A second caveat is that cells expressing TRF2ΔTR have telomeres with elongated 3ʹ overhangs [28], although there is no evidence that longer overhangs enhance the detection of t-loops [19].

### Two forms of TRF2 from a single mRNA equally protect telomeres

When human TRF2 cDNAs were originally isolated from cDNA libraries, most clones lacked the Basic N-terminus of the ORF, presumably due to the inherent problems in copying this extremely G/C-rich part of the TRF2 mRNA [2,3]. However, extensive screening of a number of cDNA libraries yielded a single TRF2 cDNA with a Kozak consensus ATG start codon [3], which was assumed to represent full-length TRF2. This cDNA and an equivalent cDNA for mouse TRF2 have been used for all in vivo and in vitro studies of TRF2 to date, including complementation of the mouse TRF2 KO phenotype [4].

**Figure 2.**
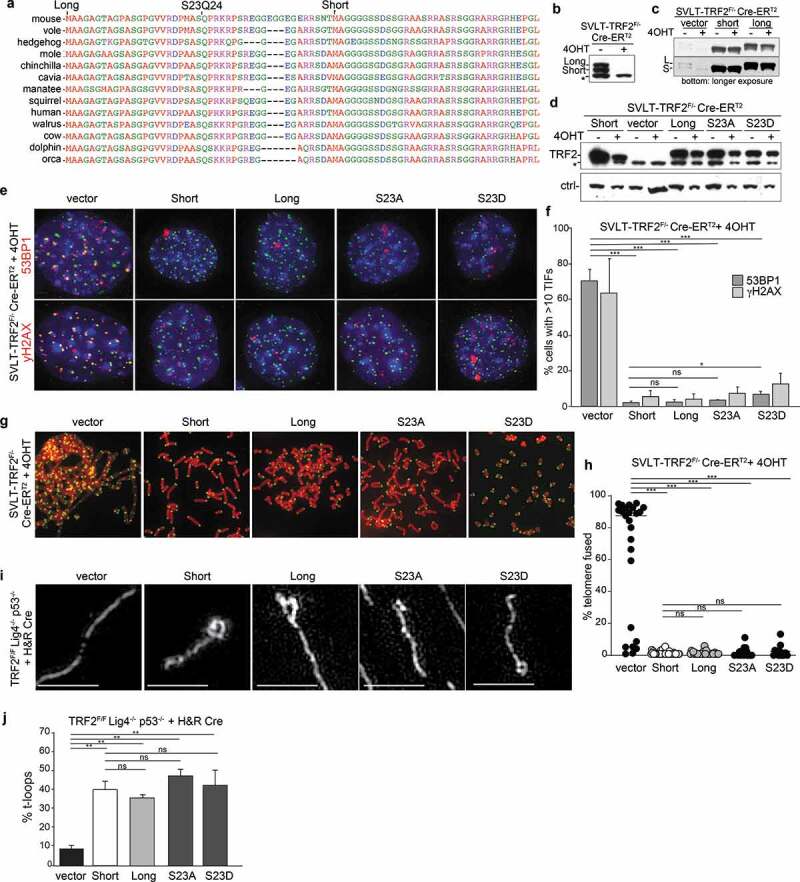
Two isoforms of TRF2 are equally proficient in telomere protection and t-loop formation. (a) Sequence alignment showing the conservation of the N-terminal sequence of TRF2. The two ATGs giving rise to the short and long form of TRF2 are indicated as well as the SQ site. Sequences were aligned using Multalign. (b) Immunoblot showing two isoforms of TRF2 that are removed upon Cre treatment of TRF2^F/F^ MEFs. (c) Immunoblot for TRF2 in the indicated MEFs with and without Cre treatment expressing the Long and Short versions of TRF2 as well as the S23A and S23D mutants of the Long form. (d) TIF assay using 53BP1 or γ-H2AX IF for detection of the telomere damage response on cells as in (c). (e) Quantification of the TIF response detected as in (d). Averages and SDs from three independent experiments with 50 nuclei each. Significance as above in Figure 1. (f) Examples of metaphase spreads with telomeres detected by FISH using the indicated MEFs infected as in (c) and treated with Cre (96 h). (g) Quantification of telomere fusions detected as in (f). Scoring was performed in three independent experiments with 10 metaphase per experiment. Averages, SDs, and significance as in Figure 1. (h) Examples of the structure of telomeric DNA detected in the indicated MEFs expressing the indicated forms of TRF2 and treated with Cre (120 h). Scale bars: 2.5 μm. (i) Quantification of t-loop frequencies detected as in (h). Averages and SDs from three independent experiments (>100 molecules scored each). Significance determined using a two-tailed unpaired t-test (n.s. not significant; ** p < 0.01).

However, according to the current database annotation (www.Ensembl.org), the human and mouse TERF2 loci express a mRNA that encodes a longer ORF that starts with a Kozak consensus ATG 42 aa of the previously identified ATG (Figure 2(a)). This N-terminal extension is highly conserved (Figure 2(a)). In immunoblots that are well resolved, our TRF2 antibody detects two bands that disappear when Cre is used to delete TRF2, potentially representing the use of both ATGs in the TRF2 mRNA (Figure 2(b)). We refer to these forms as TRF2 Long and Short. Expression of the original mouse TRF2 cDNA that starts with the second ATG results in a protein that co-migrates with TRF2 Short (Figure 2(c); Supplemental Figure 1d). To determine whether the upper band represents the full-length TRF2, we generated a construct containing the full N-terminus of TRF2 starting with the first ATG (TRF2 Long). Expression of this construct yielded a single protein that co-migrated with the Long form of TRF2 (Figure 2(c)). This result raised the question of why the second ATG in our construct is not used, whereas presumably the second ATG of the endogenous TRF2 mRNA is frequently used.

The only difference between our full-length TRF2 mRNA and the endogenous mRNA is the 5ʹ UTR. Inspection of the mouse and human 5ʹ UTRs indicates that it can form a stem-loop structure with the region surrounding the first ATG (Supplemental Figure 2a,b). Stability prediction algorithms (Sfold and mfold) showed that these sequences adopt stable folds with ΔG values of approximately −30 kcal/mol. Such secondary structure could potentially repress the use of this ATG and lead to the expression of both the Long and the Short form of TRF2 from a single mRNA. The secondary structure in the 5ʹ end of the TRF2 mRNA is highly conserved and in each case the use of the first ATG is likely to be impeded (Supplemental Figure 2a,b). If the stem-loop structure surrounding the first ATG indeed represses initiation of translation, it is predicted that initiation at the second ATG involves an IRES located upstream of the ATG. Further work will be required to identify this IRES.

Given the conserved nature of the 5ʹ end of the TRF2 mRNA and its presumed effect on the expression of the two TRF2 isoforms, it was prudent to test the ability of TRF2 Long to protect telomeres. We expressed both forms (untagged) in MEFs from which the endogenous TRF2 could be deleted with Cre (Figure 2(c)). Both forms of TRF2 were found to be equally proficient in repressing of ATM signaling at telomeres and both repressed the NHEJ of telomeres equally (Figure 2(dg)). Unsurprisingly, the two TRF2 isoforms also showed the same ability to support t-loop formation after deletion of the endogenous TRF2 (Figure 2(h,i)).

The longer isoform of TRF2 contains a conserved SQ site, the target site of the ATM and ATR kinases (Figure 2(a)). We, therefore, tested whether mutation of the Serine residue to alanine or to the phospho-mimetic glutamate affects the function of TRF2 (Figure 2(c)). Neither mutation affected the ability of TRF2 to repress ATM signaling and NHEJ (Figure 2(cg)), although the S23D mutant showed a very minor increase in TIF response. Both mutant versions of TRF2 were also capable of supporting t-loop formation in cells lacking the endogenous TRF2 (Figure 2(h,i)).

We conclude that there is no discernable difference between the ability of the two isoforms of TRF2 to protect telomeres, validating past studies in which only the shorter form of TRF2 was used for complementation and mutational analysis. Since both isoforms function equivalently at telomeres, it is unlikely that the potentially regulatory secondary structure in the TRF2 mRNA is important for telomere protection. However, there is a cytoplasmic splice variant of TRF2 (TRF2-S) that lacks the Myb DNA binding domain and the NLS. TRF2-S regulates the REST transcriptional repression complex in neuronal progenitors [32]. Potentially the two isoforms of TRF2-S that are predicted to result from differential use of the two ATGs behave differently in this regard. If so, this could explain the evolutionary conservation of the secondary structure in the 5ʹ end of the TRF2 mRNAs.

### Testing the topological model for TRF2-mediated t-loop formation

**Figure 3.**
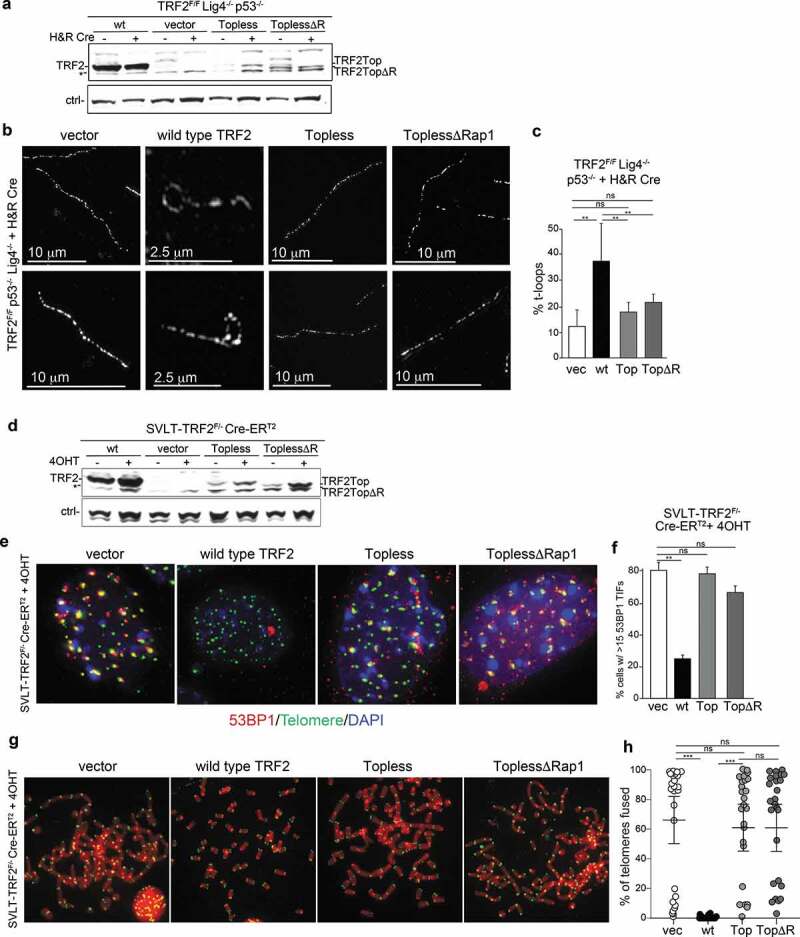
Analysis of the phenotypes associated with Topless TRF2. (a) Immunoblot for TRF2 using the indicated ligase 4-deficient MEFs with and without Cre treatment expressing wild type TRF2 (wt), the mouse version of the Topless allele with and without the Rap1 binding site (ToplessΔR). * nonspecific band. (b) Examples of the structure of telomeric DNA detected in the indicated MEFs treated with Cre (120 h) and infected as in (a). (c) Quantification of t-loop frequencies detected as in (b). Averages and SDs from three independent experiments (>100 molecules scored each). Significance determined using a two-tailed unpaired t-test (n.s. not significant; ** p < 0.01). (d) Immunoblot as in (a) but using ligase 4-proficient MEFs. (e) TIF assay using 53BP1 for detection the telomere damage response in cells as in (d). (f) Quantification of the TIF response detected as in (d). Averages and SDs from three independent experiments with 50 nuclei each. Significance as in (c). (g) Examples of metaphase spreads with telomeres detected by FISH using the indicated MEFs infected as in (d) and treated with Cre (96 h). (h) Quantification of telomere fusions detected as in (g). Scoring was performed in three independent experiments with 10 metaphase per experiment. Averages, SDs, and significance as in (c). *** p < 0.001.

To test whether TRF2 forms t-loops by changing DNA topology, we used the Topless equivalent of mouse TRF2 (containing the same 7K2 R to A mutations as in human Topless TRF2 [25]) in a setting where the endogenous TRF2 can be deleted with Cre (Figure 3). We included a version of the Topless allele that lacks the Rap1 binding site (ToplessΔR) in order to test whether the absence of Rap1 promotes telomere fusions as reported [25]. T-loop analysis showed that both Topless and ToplessΔR were severely defective in forming t-loops, consistent with prior data [25] (Figure 3(ac)). Both versions of TRF2 were also defective in the repression of ATM signaling at telomeres (Figure 3(d,e)), again consistent with prior data [25]

**Figure 4.**
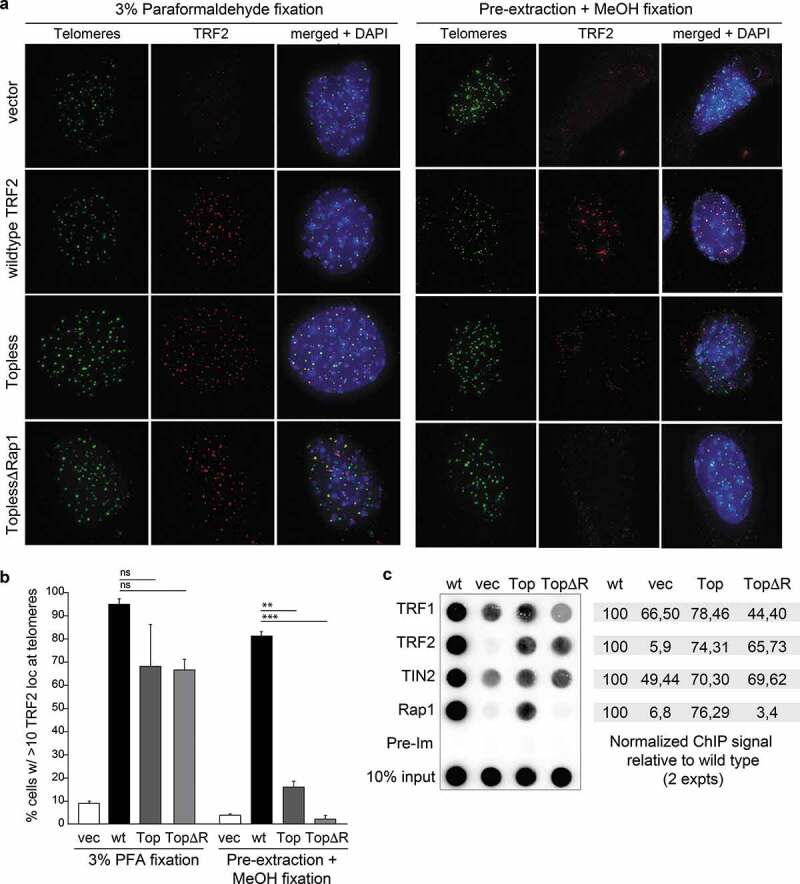
Topless TRF2 shows a defect in telomere localization. (a) IF-FISH to monitor the telomeric localization of the indicated TRF2 alleles in TRF2^F/F^ p53^-/-^ Cre-ERT^2^ MEFs at 96 h after addition of 4OHT. Left panels represent images obtained after 10 min fixation in 3% paraformaldehyde. Right panels show results obtained after extraction with Triton-X-100 followed by methanol fixation for 10 min. (b) Quantification of the localization results obtained as in (a). Averages and SDs obtained from three experiments with 100 cells each. Significance as in Figure 1. (c) ChIP using antibodies for the proteins indicated on the left using Cre-treated MEFs described in (a). Dot blot was probed for telomeric DNA. The values on the right represent the relative ChIP signals obtained with the antibodies for TRF1, TRF2, TIN2, and Rap1 in two independent experiments with the values of wild type TRF2 set at 100.

To understand why Topless TRF2 performs poorly in telomere protection, we examined its localization to telomeres more closely. Both Topless and ToplessΔR can be readily detected at telomeres in paraformaldehyde fixed samples (Figure 4(a,b)). However, when cells were pre-extracted with Triton-X100 and fixed with MeOH, these alleles show a striking deficiency in telomeric localization (Figure 4(a,b)). Quantification of the data indicates a significant reduction in the ability of Topless TRF2 to withstand pre-extraction compared to wild type TRF2, suggesting a diminished binding capacity (Figure 4(c)). ChIP analysis of formaldehyde cross-linked chromatin also showed diminished presence of the Topless alleles in association with telomeric DNA. In part, the reduced telomeric localization may be due to the lower expression level of the Topless alleles. In addition, it is not excluded that the Topless mutants have diminished or altered DNA binding activity. Although gel-shift experiments with human Topless TRF2 showed roughly the same affinity for a short telomeric substrate as the wild type TRF2, some of the R and K mutations in the TRFH domain altered the affinity [25]. Additional tests will be required to determine whether Topless TRF2 engages telomeric DNA in an altered manner.

### T-loop frequencies are similar in G1, S, and G2

**Figure 5.**
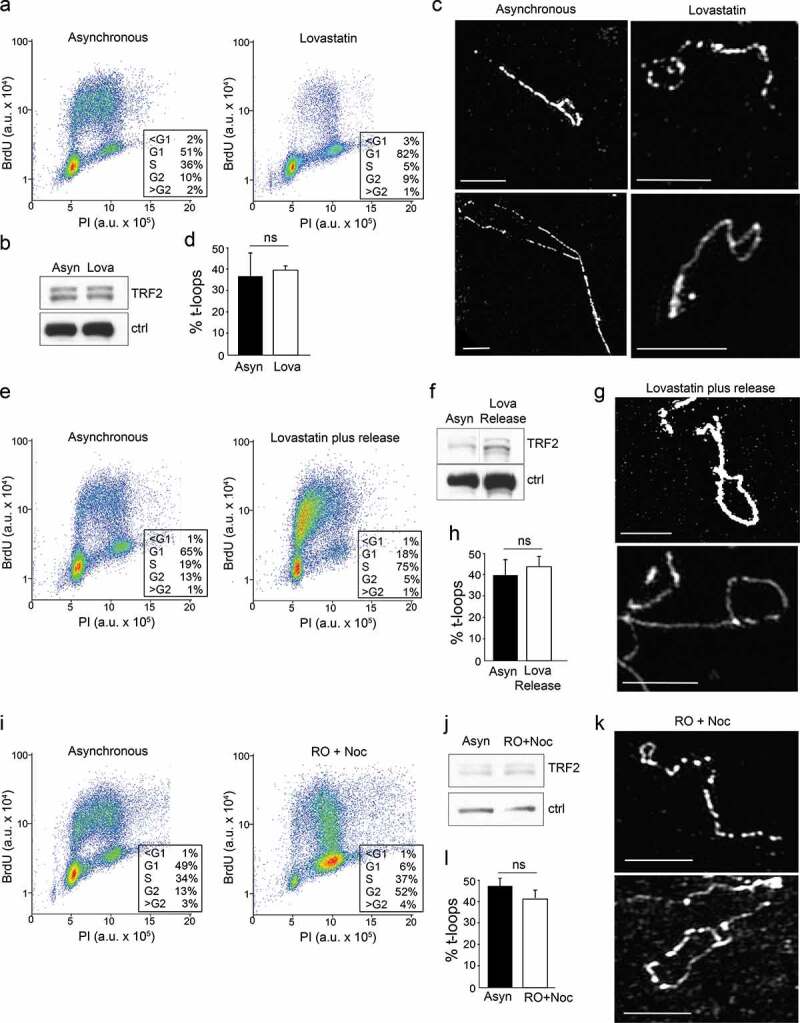
Analysis of t-loop frequencies in different cell cycle phases. (a,e,i) FACS profiles (30 min BrdU incorporation) of wild type MEFs treated to enrich cell populations in G1 (a), S (e), and S/G2 (i). For each drug regimen the asynchronous population was analyzed in parallel. (b,f,j) Immunoblots for TRF2 in each of the samples analyzed. Note that the batch of affinity purified TRF2 Ab used for these blots does not show the nonspecific band indicated in other figures. In the immunoblot in (f) a lane separating the two samples removed. Ctrl: nonspecific band used as a loading control. (c,g,k) Examples of t-loops in the indicated MEFs. Scale bars: 2.5 μm. (d,h,l) Quantification of t-loop frequencies. Averages and SEMs from two experiments per condition with 100 molecules scored for each. Significance as in Figure 1.

To gain further insight into the mechanism of t-loop formation, we asked whether this process is cell cycle regulated. The frequency of t-loops was examined in wild type MEFs that were treated with various agents to enforce enrichment in G1 (Lovastatin, Figure 5(ad)), S phase (Lovastatin followed by release into S phase, Figure 5(eg)), or G2 (RO-3306 and Nocodazole, Figure 5(il)). In each experimental setting, the cell cycle profile of the resulting cell populations was determined by FACS analysis of BrdU labeled cells. Furthermore, for each setting, we determined by immunoblotting that the expression level of TRF2 was not altered (Figure 5). Measurements of t-loop frequencies indicated the lack of a significant difference when comparing asynchronous populations to cells enriched in G1, S, or G2. We have not examined t-loop frequencies in mitosis but prior work indicates that they exist in that cell cycle stage as well [33].

The lack of cell cycle regulation of the t-loop structure precludes models for t-loop formation that invoke factors specifically expressed in S phase (e.g. certain components of the homologous recombination pathway). However, our data is not in disagreement with the idea that t-loops are resolved during the replication of telomeric DNA [34,35], since opening of t-loops would represent a short-lived state that takes place at each telomere at a different time during S phase.

The results are also informative with regard to the question of why t-loops are never observed at 100% of the telomeres analyzed. One explanation for the finding that t-loops usually are detected at less than half of the scored molecules might have been that t-loops are not present in S phase. Our data argue against this possibility although it is likely that individual t-loops are opened briefly in S phase to allow replication to proceed [34]. We consider it more likely that the t-loop frequencies are an underestimate due to breakage of telomeric DNA during the spreading technique, resulting in one linear and one t-loop molecule. Two other sources of underestimated t-loop frequencies are insufficient cross-linking at the base of the t-loop and the inability to score t-loops if the loop part is small [19].

### Rad51 is not required for t-loop formation

**Figure 6.**
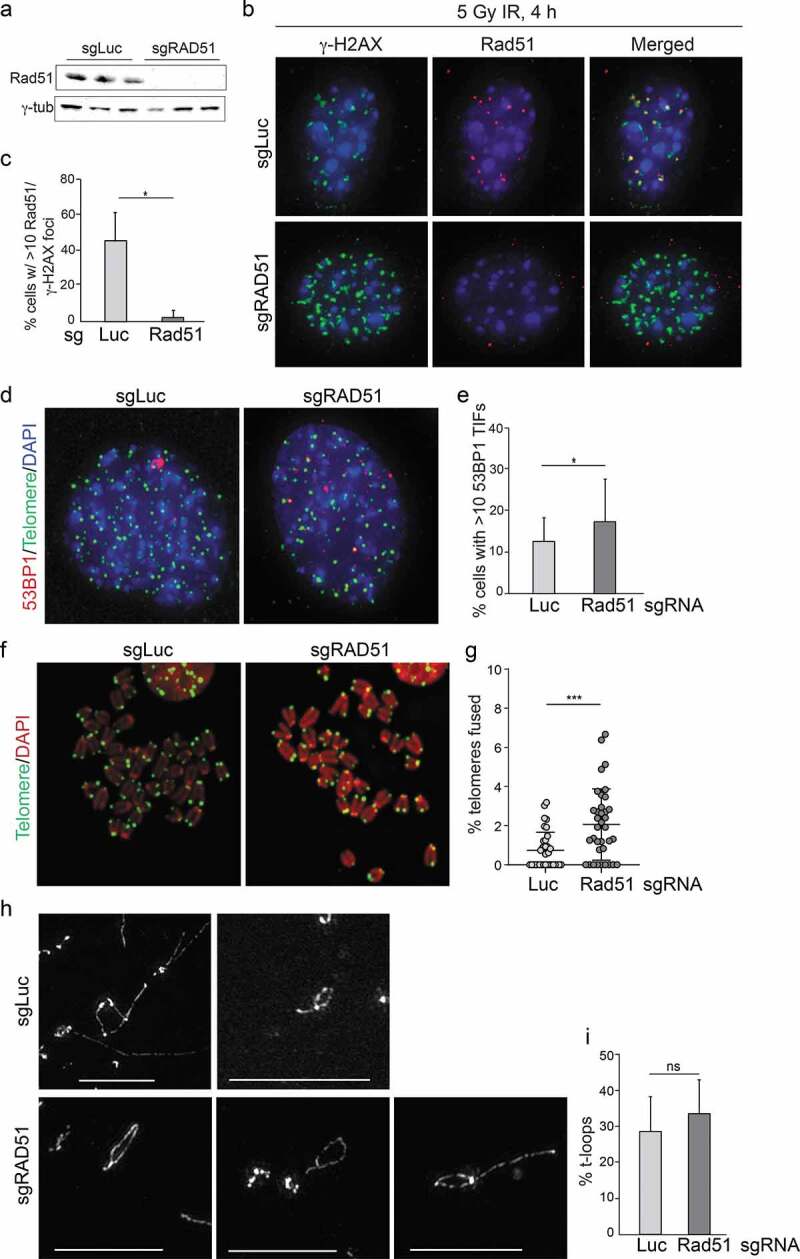
Deletion of Rad51 does induce telomere deprotection or t-loop loss. (a) Immunoblot showing loss of Rad51 expression in three population of cells treated with bulk CRISRP/Cas9 using a Rad51 sgRNA. (b) Analysis of DNA damage response foci showing absence of Rad51 at γ -H2AX foci in cells treated for Rad51 KO as in (a). Wild type MEFs were treated with a sgRNA to luciferase or to Rad51 and exposed to 4 Gy ionizing radiation (IR) 120 h later. IF for Rad51 and γ-H2AX was performed 4 h after IR. (c) Quantification of Rad51 foci detected as in (b). Averages and SDs form three experiments with 100 nuclei each. * p < 0.05 determined from a two-tailed unpaired t-test. (d) TIF analysis on cells with and without Rad51. (e) Quantification of the effect of Rad51 deletion on TIFs as in (d). Data show averages and SDs from three independent experiments. Significance as in Figure 1. (f) Examples of metaphases from cells treated with an sgRNA to Luciferase or Rad51. (g) Quantification of telomere fusions in MEFs treated with sgRNAs to Luciferase or Rad51. Data show averages and SDs from 3 experiments. Significance as in Figure 1. (h) Examples of t-loops detected in cells treated with the indicated sgRNAs as in (b). Scale bars: 2.5 μm. (i) Quantification of t-loop frequencies. Averages and SDs from three experiments with 100 molecules scored for each. Lack of significance is indicated (determined using a two-tailed unpaired t-test).

We considered that Rad51 could facilitate t-loop formation by TRF2 since this recombinase is predicted to enable the strand-invasion of the 3ʹ overhang. Indeed, Rad51 has been implicated in this role based on in vitro experiments [26]. To test the role of Rad51 we used CRISPR/Cas9 gene editing in bulk cell populations. The effective deletion of Rad51 was evaluated through immunoblotting and based on the absence of Rad51 foci in IR treated cells (Figure 6(ac)). The removal of Rad51 led to slightly elevated DNA damage response at telomeres (Figure 6(d)) and a minor increase in telomere fusions (Figure 6(e)). Both phenotypes may be due to a role for Rad51 at sites of replication stress since telomeres often experience replication problems. Nonetheless, the phenotypes of Rad51 deletion did not resemble the effects of TRF2 deletion and commensurate t-loop loss. Consistent with these findings Rad51 deletion did not alter the frequency of t-loops in the cells (Figure 6(h,i)). Although these experiments do not exclude that Rad51 contributes to t-loop formation in a redundant fashion, the data indicate that Rad51 is not required for t-loop formation.

## Conclusions

Here we show that t-loop formation requires only a single component of shelterin, TRF2. This result indicates that t-loop formation is dependent on a feature of TRF2 itself or on a second factor that is brought to telomeres by TRF2. We tested one feature of TRF2, its ability to wrap telomeric DNA, but were unable to verify that this attribute is relevant to t-loop formation due to the hypomorphic nature of the critical Topless mutant of TRF2. To better test the topology model for t-loop formation, Topless versions are needed that show normal localization to telomeres and retain the ability to repress NHEJ. We also considered the possibility that t-loop formation is not due to an intrinsic feature of TRF2 but depends on a TRF2-interacting partner. We excluded the most obvious candidate, Rad51, as being involved in t-loop formation. The extensive proteomics efforts on TRF2 complexes and telomeric chromatin have not nominated obvious additional candidates (e.g. [36–40]. Therefore, the mystery of TRF2-mediated t-loop formation remains unsolved and other models should be pursued.

## Methods

### Cell lines, retroviral gene delivery, immunoblots, ChIP analysis, TIF assays, and t-loop assays

References for the derivation and culturing of MEFs used are as follows: SV40 Large T immortalized (SVLT)-TRF2^F/-^ Cre-ERT^1^ [19]; TRF2^F/F^ Lig4^-/-^ p53^-/-^ [27]; TRF1^F/F^ TRF2^F/F^ Lig4^-/-^ p53^-/-^ Cre-ERT^2^ [27]; TRF1^F/F^ TRF2^F/F^ Ku80^-/-^ p53^-/-^ Cre-ERT^2^ [27]. Retroviral infections were performed as described [27]. Cre-mediated deletion was induced with either two infections of pMMP Hit&Run Cre retrovirus over 24 h, or by inducing Cre-ER^T2^ expressed from the Rosa26 locus with 0.5 µM tamoxifen (4OHT, Sigma H7904) for 12 h. Time 0 was set at the media exchange after tamoxifen induction or the second viral infection. Immunoblots were performed as described [27] using antibody 1254 for TRF2 and an antibody for mouse Rad51 provided by Roland Kanaar (Univ. Erasmus, Rotterdam) and an antibody to γ-tubulin (GT488, Sigma). Telomeric ChIP was performed as described previously [27] using the following antibodies: mouse TRF2, 1254; mouse TRF1, 1449; mouse Rap1, 1251; mouse TIN2, 1447. IF and IF-FISH was performed as described [27] using Abcam ab175933 for 53BP1, Millipore 05–636 for γ-H2AX, mouse TRF2 antibody 1254, and the mouse Rad51 Ab described above. T-loop assays were performed as originally described by Doksani et al. [19] with modifications as described [31] and imaged using a GE OMX V4. Imaging of TIFs and metaphase spreads was performed on a Zeiss Axioplan II with a 63X objective lens and a Hamamatsu C4742-95 and processed with Volocity or on a GE Deltavision with a 60X objective and processed with FIJI.

### Altered forms of mouse TRF2

The version of TRF2 lacking the Rap1 and TIN2 bindings sites (ΔTR, Δ350-365 [41] and Δ284-297 [29]) was generated by PCR-based mutagenesis on a version of TRF2 lacking the Basic domain and then cloned into a pLPC vector containing the Basic domain with an N-terminal Myc tag and C-terminal BamHI site. For generating the long version of TRF2, the whole N-terminal sequence (including the Basic domain) was synthesized by Genewiz and cut out of a plasmid with BglII and BamHI. This fragment was ligated to the BamHI site of a pLPC plasmid containing the rest of TRF2 with a BamHI site at the N-terminus of the TRFH domain [31]. Although both ATGs are present in this construct, only the first ATG appears to be used in vivo. The TRF2 Topless mutant was generated by site-directed mutagenesis with the following aa changed to alanine: R69, R99, K158, K173, K176, K179, K241, K242, and K245. Mutagenesis was performed on mTRF2 lacking the Basic domain in pBluescript. The mutant version was then moved into a pLPC vector containing the Basic domain with an N-terminal Myc tag and a C-terminal BamHI site. This Topless version was used to delete the Rap1 binding site (Δ284-297) by PCR-based mutagenesis.

### Cell cycle experiments

MEFs were treated with either 9 µM RO-3306 (Sigma, SML0569-5 M) for 12 h and released into 1 µg/ml Nocadozole (Sigma, M1404) for 2 h, or with 40 µM Lovastatin (Apex Bio A4365) for 36 h, or with 40 µM Lovastatin for 36 h and released into 400 µM Mevalonic Acid (Sigma, 90469–10 MG) for 16 h, or with DMSO as a control. Flow cytometry was conducted on an Accuri C6 and cells were prepared as follows: 1 h prior to harvest cells were treated with 10 µM BrdU and 500,000 cells were then harvested and fixed overnight in −20°C 70% EtOH. Cells were denatured and permeabilized with 2 N HCl/0.5% Triton X-100 for 30 minutes, then neutralized with 0.1 M Sodium Borate. Cells were blocked with 0.5% BSA/0.5% Tween-20 in PBS and incubated with a FITC-conjugated anti-BrdU antibody (BD Biosciences #347583), then resuspended in 2 mM EDTA, 0.5 μg/ml RNAse A, and 5 µg/ml Propidium Iodine for FACS analysis. The data were processed with Flojo software.

### CRISPR/Cas9 deletion of Rad51

Rad51 was targeted using a lentiviral vector expressing Cas9 and simple guide RNAs (Lenti CRISPRv2, Addgene #52961). The sgRNA guide sequence for Rad51 was: 5ʹ-AGCCTCCACTGTATGGTAAC-3ʹ. The control sgRNA to Luciferase had the guide sequence: 5ʹ-ACAACTTTACCGACCGCGCC-3ʹ. Viral production and infections were performed as described [42].

## Supplementary Material

Supplemental Material

## References

[1] de Lange T. Shelterin-mediated telomere protection. Annu Rev Genet. 2018;52:223–247. 30208292 10.1146/annurev-genet-032918-021921

[2] Bilaud T, Brun C, Ancelin K, et al. Telomeric localization of TRF2, a novel human telobox protein. Nat Genet. 1997;17:236–239. 9326951 10.1038/ng1097-236

[3] Broccoli D, Smogorzewska A, Chong L, et al. Human telomeres contain two distinct Myb-related proteins, TRF1 and TRF2. Nat Genet. 1997;17:231–235. 9326950 10.1038/ng1097-231

[4] Celli GB, De Lange T. DNA processing is not required for ATM-mediated telomere damage response after TRF2 deletion. Nat Cell Biol. 2005;7:712–718. 15968270 10.1038/ncb1275

[5] Celli GB, Lazzerini Denchi E, de Lange T. Ku70 stimulates fusion of dysfunctional telomeres yet protects chromosome ends from homologous recombination. Nat Cell Biol. 2006;8:885–890. 16845382 10.1038/ncb1444

[6] Karlseder J, Broccoli D, Dai Y, et al. p53- and ATM-dependent apoptosis induced by telomeres lacking TRF2. Science. 1999;283:1321–1325. 10037601 10.1126/science.283.5406.1321

[7] Martinez P, Thanasoula M, Munoz P, et al. Increased telomere fragility and fusions resulting from TRF1 deficiency lead to degenerative pathologies and increased cancer in mice. Genes Dev. 2009;23:2060–2075. 19679647 10.1101/gad.543509 PMC2751970

[8] Sfeir A, Kosiyatrakul ST, Hockemeyer D, et al. Mammalian telomeres resemble fragile sites and require TRF1 for efficient replication. Cell. 2009;138:90–103. 19596237 10.1016/j.cell.2009.06.021 PMC2723738

[9] van Steensel B, Smogorzewska A, de Lange T. TRF2 protects human telomeres from end-to-end fusions. Cell. 1998;92:401–413. 9476899 10.1016/s0092-8674(00)80932-0

[10] Li B, Oestreich S, de Lange T. Identification of human Rap1: implications for telomere evolution. Cell. 2000;101(5):471–483. 10850490 10.1016/s0092-8674(00)80858-2

[11] Liu D, O’Connor MS, Qin J, et al. Telosome, a mammalian telomere-associated complex formed by multiple telomeric proteins. J Biol Chem. 2004a;279:51338–51342. 15383534 10.1074/jbc.M409293200

[12] Ye JZ, Donigian JR, Van Overbeek M, et al. TIN2 binds TRF1 and TRF2 simultaneously and stabilizes the TRF2 complex on telomeres. J Biol Chem. 2004a;279:47264–47271. 15316005 10.1074/jbc.M409047200

[13] Houghtaling BR, Cuttonaro L, Chang W, et al. A dynamic molecular link between the telomere length regulator TRF1 and the chromosome end protector TRF2. Curr Biol. 2004;14:1621–1631. 15380063 10.1016/j.cub.2004.08.052

[14] Liu D, Safari A, O’Connor MS, et al. PTOP interacts with POT1 and regulates its localization to telomeres. Nat Cell Biol. 2004b;6:673–680. 15181449 10.1038/ncb1142

[15] Ye JZ, Hockemeyer D, Krutchinsky AN, et al. POT1-interacting protein PIP1: a telomere length regulator that recruits POT1 to the TIN2/TRF1 complex. Genes Dev. 2004b;18:1649–1654. 15231715 10.1101/gad.1215404 PMC478187

[16] Baumann P, Cech TR. Pot1, the putative telomere end-binding protein in fission yeast and humans. Science. 2001;292:1171–1175. 11349150 10.1126/science.1060036

[17] Denchi EL, de Lange T. Protection of telomeres through independent control of ATM and ATR by TRF2 and POT1. Nature. 2007;448:1068–1071. 17687332 10.1038/nature06065

[18] Kibe T, Osawa GA, Keegan CE, et al. Telomere protection by TPP1 is mediated by POT1a and POT1b. Mol Cell Biol. 2010;30:1059–1066. 19995905 10.1128/MCB.01498-09 PMC2815557

[19] Doksani Y, Wu JY, De Lange T, et al. Super-resolution fluorescence imaging of telomeres reveals TRF2-dependent T-loop formation. Cell. 2013;155:345–356. 24120135 10.1016/j.cell.2013.09.048 PMC4062873

[20] Griffith JD, Comeau L, Rosenfield S, et al. Mammalian telomeres end in a large duplex loop. Cell. 1999;97:503–514. 10338214 10.1016/s0092-8674(00)80760-6

[21] Bae NS, Baumann P. A RAP1/TRF2 complex inhibits nonhomologous end-joining at human telomeric DNA ends. Mol Cell. 2007;26:323–334. 17499040 10.1016/j.molcel.2007.03.023

[22] Gaullier G, Miron S, Pisano S, et al. A higher-order entity formed by the flexible assembly of RAP1 with TRF2. Nucleic Acids Res. 2016;44:1962–1976. 26748096 10.1093/nar/gkv1531 PMC4770236

[23] Okamoto K, Bartocci C, Ouzounov I, et al. A two-step mechanism for TRF2-mediated chromosome-end protection. Nature. 2013;494:502–505. 23389450 10.1038/nature11873 PMC3733551

[24] Fairall L, Chapman L, Moss H, et al. Structure of the TRFH dimerization domain of the human telomeric proteins TRF1 and TRF2. Mol Cell. 2001;8:351–361. 11545737 10.1016/s1097-2765(01)00321-5

[25] Benarroch-Popivker D, Pisano S, Mendez-Bermudez A, et al. TRF2-mediated control of telomere DNA topology as a mechanism for chromosome-end protection. Mol Cell. 2016;61:274–286. 26774283 10.1016/j.molcel.2015.12.009 PMC5001171

[26] Verdun RE, Karlseder J. The DNA damage machinery and homologous recombination pathway act consecutively to protect human telomeres. Cell. 2006;127:709–720. 17110331 10.1016/j.cell.2006.09.034

[27] Sfeir A, de Lange T. Removal of shelterin reveals the telomere end-protection problem. Science. 2012;336:593–597. 22556254 10.1126/science.1218498 PMC3477646

[28] Kibe T, Zimmermann M, de Lange T. TPP1 blocks an ATR-mediated resection mechanism at telomeres. Mol Cell. 2016;61:236–246. 26778124 10.1016/j.molcel.2015.12.016 PMC4724337

[29] Sfeir A, Kabir S, van Overbeek M, et al. Loss of Rap1 induces telomere recombination in the absence of NHEJ or a DNA damage signal. Science. 2010;327:1657–1661. 20339076 10.1126/science.1185100 PMC2864730

[30] Takai KK, Kibe T, Donigian JR, et al. Telomere protection by TPP1/POT1 requires tethering to TIN2. Mol Cell. 2011;44:647–659. 22099311 10.1016/j.molcel.2011.08.043 PMC3222871

[31] Schmutz I, Timashev L, Xie W, et al. TRF2 binds branched DNA to safeguard telomere integrity. Nat Struct Mol Biol. 2017;24:734–742. 28805810 10.1038/nsmb.3451

[32] Grammatikakis I, Zhang P, Mattson MP, et al. The long and the short of TRF2 in neurogenesis. Cell Cycle. 2016;15:3026–3032. 27565210 10.1080/15384101.2016.1222339 PMC5134711

[33] Van Ly D, Low RRJ, Frölich S, et al. Telomere loop dynamics in chromosome end protection. Mol Cell. 2018;71:510525.e6. DOI:10.1016/j.molcel.2018.06.025 30033372

[34] Sarek G, Vannier JB, Panier S, et al. TRF2 recruits RTEL1 to telomeres in S phase to promote t-loop unwinding. Mol Cell. 2015;57:622–635. 25620558 10.1016/j.molcel.2014.12.024 PMC4339303

[35] Vannier JB, Pavicic-Kaltenbrunner V, Petalcorin MI, et al. RTEL1 dismantles T loops and counteracts telomeric G4-DNA to maintain telomere integrity. Cell. 2012;149:795–806. 22579284 10.1016/j.cell.2012.03.030

[36] Bartocci C, Diedrich JK, Ouzounov I, et al. Isolation of chromatin from dysfunctional telomeres reveals an important role for Ring1b in NHEJ-mediated chromosome fusions. Cell Rep. 2014;7:1320–1332. 24813883 10.1016/j.celrep.2014.04.002 PMC4054697

[37] Dejardin J, Kingston RE. Purification of proteins associated with specific genomic Loci. Cell. 2009;136:175–186. 19135898 10.1016/j.cell.2008.11.045 PMC3395431

[38] Gao XD, Tu L-C, Mir A, et al. C-BERST: defining subnuclear proteomic landscapes at genomic elements with dCas9-APEX2. Nat Methods. 2018;15:433–436. 29735996 10.1038/s41592-018-0006-2 PMC6202229

[39] Garcia-Exposito L, Bournique E, Bergoglio V, et al. Proteomic profiling reveals a specific role for translesion DNA polymerase η in the alternative lengthening of telomeres. Cell Rep. 2016;17:1858–1871. 27829156 10.1016/j.celrep.2016.10.048 PMC5406014

[40] Porreca RM, Herrera-Moyano E, Skourti E, et al. TRF1 averts chromatin remodelling, recombination and replication dependent-break induced replication at mouse telomeres. Elife. 2020;9. DOI:10.7554/eLife.49817 PMC6986873 31934863

[41] Takai KK, Hooper S, Blackwood S, et al. In vivo stoichiometry of shelterin components. J Biol Chem. 2010;285:1457–1467. 19864690 10.1074/jbc.M109.038026 PMC2801271

[42] Mirman Z, Lottersberger F, Takai H, et al. 53BP1-RIF1-shieldin counteracts DSB resection through CST- and Polα-dependent fill-in. Nature. 2018;560:112–116. 30022158 10.1038/s41586-018-0324-7 PMC6072559

