# Supplementary material for: Characterization of t-loop formation by TRF2
Source: Nucleus. 2020 Jul 14;11(1):164–77. doi: 10.1080/19491034.2020.1783782 (PMC7529409; doi:10.1080/19491034.2020.1783782)
Supplement: Supplemental Material [file KNCL_A_1783782_SM0931.zip › Supplementary information/Supplemental Figure legends.docx]

**Supplemental Figure 1. TRF2 is sufficient for t-loop formation.**

**a**, Immunoblot for the endogenous and exogenous TRF2 expressed in the indicated MEFs, illustrating the greater abundance of the exogenously expressed TRF2. *, non-specific band detected in the overloaded +Cre lane. Ctrl, non-specific band used as loading control. **b**, Immunblot for TRF2 in the indicated Ku80-deficient MEFs expressing wild type TRF2 (wt), TRF2ΔTR (ΔTR), or no exogenous TRF2 (vec) after induction of Cre with 4-OHT. **b**, Examples of telomeric DNA structure in the indicated cells. **c**, Quantification of t-loop frequencies as in Figure 1d.

**Supplemental Figure 2. A conserved structure in the 5’ end of the TRF2 mRNA.**

**a**, The 5’ ends of the TRF2 mRNA from the indicated species (AUG highlighted in red) are shown together with their secondary structure predictions according to RNAfold (<http://rna.tbi.univie.ac.at/cgi-bin/RNAWebSuite/RNAfold.cgi>). The annotated 5’ ends of the TRF2 mRNAs from cow and sperm whale are assumed to be wrong since a highly conserved sequence (green) is present immediately upstream. The secondary structure predictions were done with the presumed mRNA starting at the same site as in human and mouse. **b**, Predicted secondary structures of the four mRNAs displayed using the VARNA applet (http://varna.lri.fr/). The ATGs are highlighted. ΔGs are greater than -30 kcal/mol for each structure based on mfold and Sfold.
