# Supplementary figures and images for: Characterization of t-loop formation by TRF2
Source: Nucleus. 2020 Jul 14;11(1):164–77. doi: 10.1080/19491034.2020.1783782 (PMC7529409; doi:10.1080/19491034.2020.1783782)

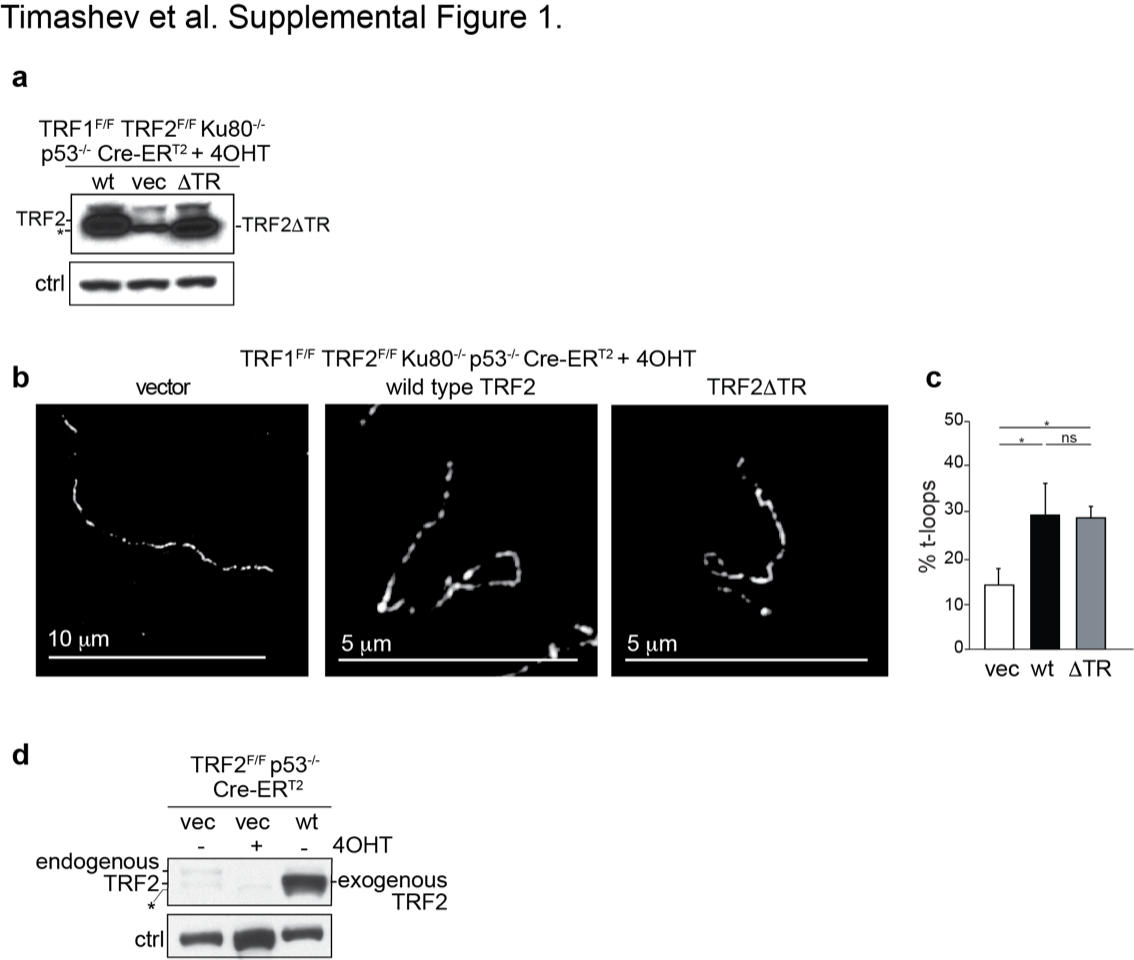

Supplement: Supplemental Material [file KNCL_A_1783782_SM0931.zip › Supplementary information/Supplemental_Figure_1_Leonid.png]

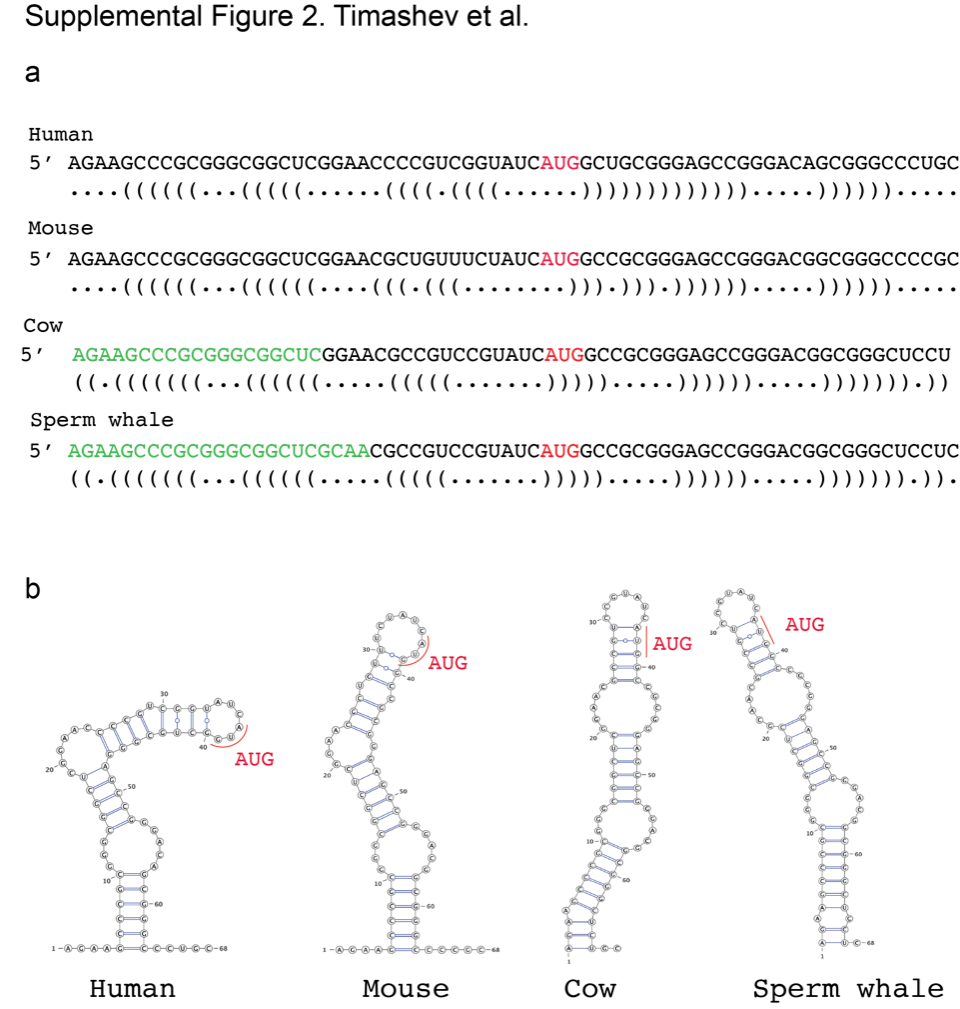

Supplement: Supplemental Material [file KNCL_A_1783782_SM0931.zip › Supplementary information/Supplemental_Figure_2.png]
